# Biochemical Control in a Colombian Cohort of Patients With Acromegaly: A 12-Month Follow-Up Study (2017-2023)

**DOI:** 10.7759/cureus.75553

**Published:** 2024-12-11

**Authors:** Alin Abreu Lomba, David Corredor-Rengifo, Cesar Augusto Mejia Velez, Reynaldo Carvajal Ortiz, Doly Pantoja Guerrero, Henry Mauricio Arenas, Alejandro Alberto Castellanos Pinedo, Monica Andrea Morales Garcia, Alejandro Pinzon Tovar, David Alexander Vernaza Trujillo, Santiago Sierra Castillo

**Affiliations:** 1 Endocrinology, Imbanaco Clinic, Cali, COL; 2 Internal Medicine, Universidad Libre, Cali, COL; 3 Epidemiology and Biostatistics, Universidad Libre, Cali, COL; 4 Endocrinology, Departmental University Hospital of Nariño Pasto, Pasto, COL; 5 Endocrinology, Clínica Comfamiliar Pereira, Pereira, COL; 6 Endocrinology, Hospital Escuela Jose de San Martín, Buenos Aires, ARG; 7 Epidemiology and Biostatistics, Imbanaco Clinic, Cali, COL; 8 Endocrinology, Universidad Surcolombiana, Neiva, COL; 9 Internal Medicine, ENDHO Colombia, Neiva, COL; 10 Epidemiology, Fundación Universitaria Del Área Andina, Bogotá, COL; 11 Interinstitutional Group of Internal Medicine, Universidad Libre, Universidad Libre, Cali, COL; 12 Epidemiology, CES University, Medellín, COL

**Keywords:** acromegaly and surgery, growth hormone secreting pituitary adenoma, insulin-like growth factor 1, macro adenoma, pituitary tumour

## Abstract

Background: Acromegaly, although rare, is associated with multiple manifestations and complications; its high morbidity and mortality makes it a challenge. Treatment involves surgery and pharmacological therapies, focusing on biochemical normalization. This study analyzes the biochemical control in Colombian patients with acromegaly, seeking to improve the understanding of the effects of treatments in the management of the disease.

Methods: A multicenter retrospective cohort study was conducted with data from a national acromegaly registry in Colombia (2017-2023), analyzing the biochemical control for 12 months according to the treatment modalities received.

Results: A total of 117 patients were analyzed, with 54 individuals from Valle del Cauca and 63 being women, representing different population groups in Colombia. The median age was 52 years, and the median disease duration was six years. Clinically, arterial hypertension and sleep apnea were observed in 53.8% (n = 63) and 45.3% (n = 53) of the cohort, respectively. Biomarker analysis revealed elevated levels of insulin-like growth factor-1 (IGF-1) and growth hormone (GH). The majority of tumors were macroadenomas, and among the 103 surgically removed tumors, all secreted GH. Of these, 58.3% (n = 60) had GH as the sole marker, while 12.6% (n = 13) co-expressed prolactin (PRL). At first, 92.3% (n = 108) of patients had no biochemical control. At six and 12 months, 34.1% (n = 40) and 21.2% (n = 25) achieved biochemical control, respectively. The reduction in tumor size was significant during follow-up, with a median size at the month of admission of 16 mm, with a reduction >20% at month 12 in 92.3% (n = 108) of patients.

Conclusion: In Colombian patients with acromegaly, biochemical control at 12 months is lower than that reported in the literature, suggesting that pharmacological management could be associated with greater biochemical control.

## Introduction

Acromegaly is a secondary entity to the overproduction of growth hormone (GH), in more than 95% of cases due to a pituitary tumor; the final consequence of this overproduction of GH is the induction of insulin-like growth factor 1 (IGF-1), which is the molecule that generates the peripheral effect in tissues and systems, producing important metabolic and morphological changes in the patient [1]. It is a rare disease, with an estimated prevalence of 2.8 cases per 100,000 people/year, and an incidence between 0.1 and 1.1 cases per 100,000 people/year [2]; however, it has a high burden of disease and both cardiovascular and non-cardiovascular complications, which increase mortality compared to the general population [3].

In Colombia, due to challenges in data collection, it is difficult to accurately estimate the true prevalence of acromegaly. Using indirect calculations, the prevalence rate is estimated at 27.61 cases per million affiliated individuals [4]. The identification and correct treatment of this pathology should be a priority for health professionals, but this is hindered by non-specific symptoms at the beginning of the disease, leading to a late diagnosis [1].

There are several management modalities included in guidelines and consensus, which propose surgical treatment as the first line of management, pharmacological treatments and radiotherapy, considered second-line and adjunctive therapies [5,6]. After surgery, the biochemical response of the disease should be determined with IGF-1 and GH measurement three months after the intervention [6]. The primary treatment goal is biochemical normalization of IGF-1 levels less than 1.2 times the upper limit for age and achieving a GH value less than 1 ng/mL in a random sample [5,6]. Magnetic resonance imaging (MRI) in the brain should also be performed three months after surgery and is considered a reduction in tumor size when it is greater than 20% [1,5]. A controlled patient is defined as meeting both biochemical and imaging criteria. Patients who do not reach biochemical goals need other treatment alternatives, such as repeat surgery, pharmacological therapy, or radiation therapy [1,5-7].

The need for effective treatments that achieve biochemical control has led to a greater use of complementary strategies to surgical management, such as the use of somatostatin analogues (SSA), reducing mortality, as opposed to patients who are subjected to surgical management alone with or without radiotherapy [8]. In recent decades, this has led to higher rates of biochemical control and a decrease in mortality, possibly with greater use of SSA or other adjuvant pharmacological therapies [9].

In Colombia, there is a lack of studies assessing the impact of different treatments on the progression of acromegaly and their influence on biochemical control. Addressing this gap is crucial to optimize healthcare resources and implement individualized treatment strategies. This study aims to evaluate the biochemical control in a cohort of Colombian patients with acromegaly, followed for 12 months, based on the treatments received during the period 2017-2023.

## Materials and methods

Study design and patient selection

A multicenter retrospective cohort study was conducted with patients enrolled in the National Acromegaly Registry (RAPACO) [10], to analyze the biochemical control achieved with different therapies offered during one year of follow-up, in the period 2017 - 2023. A census included all patients diagnosed with acromegaly, over 18 years of age, who had complete information on biochemical GH measurements, IGF-1, and tumor size measurement by Empty Sella MRI at baseline and follow-up at six and 12 months. Patients who underwent surgery and did not have histopathology results of the specimen extracted were excluded.

Definition of variables and data collection

The main exposure variable was the types of treatment received at the beginning of the observation, at six and 12 months; with which three groups were defined for the analysis: patients who received surgical management, patients who received surgical management plus pharmacological management, and patients who received pharmacological management alone. In addition, in each period, the active intervention received (surgical, pharmacological, and pharmaco-surgical) was evaluated, as well as cumulative intervention (those patients taken to surgery were grouped into the surgical or pharmaco-surgical group in all periods after the intervention).

The study event was the biochemical control observed during the 12-month follow-up and defined as those patients who achieved IGF-1 levels less than 1.2 times the age-adjusted upper limit of normal and ultrasensitive GH values less than 1 ng/mL. Given the above, three possible outcomes were found: controlled patients who met both conditions, patients with partial control who only achieved adequate levels of one of the biomarkers, and uncontrolled patients. Other variables of clinical interest, such as age, signs, and symptoms of the disease, were also collected. The data were taken from RAPACO, which has collected information on patients with the disease since 2017, from 14 departments in Colombia.

Statistical analysis

A description of the clinical characteristics of the selected population was carried out by constructing absolute and relative frequency tables with categorical variables and comparing the proportions obtained with the Chi2 or Fisher test. The quantitative variables were summarized with medians and interquartile ranges (IQR) depending on the distribution, exploring their relationship with the Mann-Whitney U test. The proportion of the biochemical control categories was estimated in contrast to the treatments offered during the months of follow-up and compared using the Z-test. To determine the possible differences in biochemical control between months six and 12 of follow-up in relation to month zero, the McNemar test was used. All calculations were performed using 95% confidence intervals. A value of p <0.05 was statistically significant. The analyses were run in STATA software version 15.1 ® (Stata-Corp, College Station, TX, USA) and in Microsoft Excel® (Redmond, WA, USA).

Ethical considerations

The protocol of this research under the project code CI-02-21-09 was reviewed and approved by the research and ethics committee of the Universidad de Libre de Cali through Minute No. 01 of February 21, 2024. The protocol coded as CEI-878 by the Imbanaco Clinic was also reviewed and approved by code CEI-202 of March 15, 2024. It was considered a minimal-risk investigation according to resolution 8430 of 1993 of the Colombian Ministry of Health, Resolution No. 2378 of 2008, of the Ministry of Social Protection of Colombia, the Guide to Good Clinical Practices (ICH-GCP E6), as well as its adherence to the principles of the World Medical Assembly set forth in the Declaration of Helsinki of 1964.

## Results

**Figure 1:**
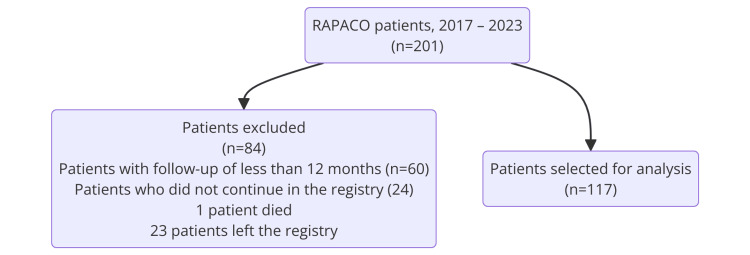
Diagram of the selection of the population studied. RAPACO: Registry of Acromegalic Patients in Colombia

A total of 201 patients were found in the RAPACO registry during the study period. A total of 117 patients were included according to the selection criteria (Figure 1).

Cohort description and disease manifestations

The selected population consisted of 117 patients, with 54 (46.1%) from the Valle del Cauca region and 63 (53.8%) women. The median age of the cohort was 52 years (IQR, 40 to 60). The median time of disease evolution was six years (IQR, 4 to 8 years) until entry into the registry (Table 1). In the description of the clinical findings, it was found that arterial hypertension was the most frequent cardiovascular manifestation, followed by obstructive sleep apnea. Among neuropsychiatric manifestations, headache and visual disturbances were commonly reported. Musculoskeletal manifestations were represented by carpal tunnel syndrome and fatigue (Table 2). Almost half of the patients had hyperprolactinemia as the main endocrinological manifestation (n=56, 47.9%) while hypopituitarism was reported in n=36 (30.8%) of the subjects (Table 3). Adenomatous polyps were the main gastrointestinal finding (n=13, 11.1%) and growth of acral areas was the most relevant systemic manifestation of the disease. There were no statistically significant differences in clinical findings between men and women.

**Table 1:** Clinical Characteristics of Selected Patients Md: Median, IQR: Interquartile Range

Characteristics	Total (n=117)
Age in years, Md (IQR)	52 (40-60)
Women, n (%)	63 (53.85)
Valle del Cauca	54 (46.15)
Atlantico	21 (17.84)
Santander	17 (9.60)
Huila	13 (11.11)
Other	12 (10.25)
Disease duration in years, Md (IQR)	6 (4-8)

**Table 2:** Clinical Characteristics

Clinical manifestations by systems	n (%)
Hypertension	63 (53.85)
Obstructive sleep apnea	53 (45.30)
Cardiomyopathy	41 (35.04)
Cardiac arrhythmias	25 (21.37)
Headache	15 (12.82)
Visual disturbances	8 (6.84)
Decreased libido	6 (5.13)
Depression	8 (6.84)
Blindness	3 (2.56)
Carpal tunnel syndrome	23 (19.66)
Fatigue	14 (11.97)
Arthralgia	11 (9.4)
Hand dysesthesias	5 (4.27)
Constipation	8 (6.84)
Hand growth	81 (69.23)
Foot growth	72 (61.54)
Facial growth	66 (56.41)
Hyperhidrosis	4 (3.42)

**Table 3:** Endocrine Characteristics

Endocrine Characteristics	n (%)
Hyperprolactinemia	56 (47.86)
Hypopituitarism	36 (30.77)
Diabetes Mellitus	27 (23.08)
Adenomatous polyps	13 (11.11)
Hyperplastic polyps	12 (10.26)

Biomarkers of acromegaly at the time of entry into the registry showed elevated levels of IGF-1 and GH with no differences by sex (IGF-1 800 ng/mL; RIC, 517 to 1076 ng/mL, and GH 14 ng/mL; IQR, 5.85 to 25 ng/mL, respectively). Most tumors were classified as macroadenomas by imaging (80.3%, n=94) (Tables 4, 5).

**Table 4:** Baseline biochemistry Md: Median, IQR: Interquartile Range, IGF-1: Insulin-like Growth Factor 1, GH: Growth Hormone

Baseline biochemistry	Md (IQR)
IGF-1 (ng/mL)	800 (517.5-1076)
GH (ng/mL)	14 (5.85-25)
Glucose (mg/dL)	96 (86-113.5)
Prolactin (ng/mL)	19 (10-31.5)

**Table 5:** Tumor characteristics Md: Median, IQR: Interquartile Range, MRI: Magnetic Resonance Imaging.

Tumor characteristics	
Tumor size in mm by MRI, Md (IQR)	16 (12-19)
Microadenoma n %	23 (19.66)
Macroadenoma n%	94 (80.34)

Tumor characteristics

The immunohistochemical and cellular characteristics of the tumors removed from 103 patients during the study period were studied (Table 6), in a total of 158 surgeries. The median mitotic activity measured through the Ki67% protein was 2%; however, 30 specimens showed a Ki67% activity greater than 3%, distributed in a total of 19 patients. There was low expression of the p53 protein in the tumors. Cell lineage profiles showed that all tumors expressed GH, in addition, it was expressed as a single marker in n=60 (58.3%) of the cases, it was also associated with combinations with other cell lineages since n=13 (12.6%) of the tumors expressed GH and prolactin (PRL) while n=3 (2.9%) expressed GH, PRL and some other marker. 

**Table 6:** Immunohistochemical Characteristics of Tumors in Patients Undergoing Surgical Resection GH: Growth Hormone, PRL: Prolactin, FSH: Follicle-Stimulating Hormone, ACTH: Adrenocorticotropic Hormone, TSH: Thyroid-Stimulating Hormone, LH: Luteinizing Hormone.

Characteristic	Total (n=103)	Microadenoma (n=15)	Macroadenoma (n=88)	p-value
Ki67 (%), Md (IQR)	2 (0-5)	1 (0-6)	2 (0-5)	0.74
p53 (%), Md (IQR)	0 (0-1)	0 (0-3)	0 (0-1)	0.61
GH	103 (100)	15 (100)	88 (100)	-
PRL	17 (16.50)	1 (6.67)	16 (18.18)	0.46
FSH	6 (5.83)	1 (6.67)	5 (5.68)	1.00
ACTH	5 (5.05)	1 (7.14)	4 (4.71)	0.54
TSH	4 (4.49)	1 (8.33)	3 (3.90)	0.45
LH	2 (1.94)	1 (6.67)	1 (1.14)	0.27
GH-only expression	60 (58.25)	8 (53.33)	52 (59.09)	0.77
Combined GH and PRL expression	13 (12.62)	0 (0)	13 (14.77)	0.00
Combined GH and others expression	11 (10.68)	2 (13.33)	9 (10.23)	0.66
Combined GH, PRL, and others expression	3 (2.91)	1 (6.67)	2 (2.27)	0.37

Interventions and follow-up

**Figure 2:**
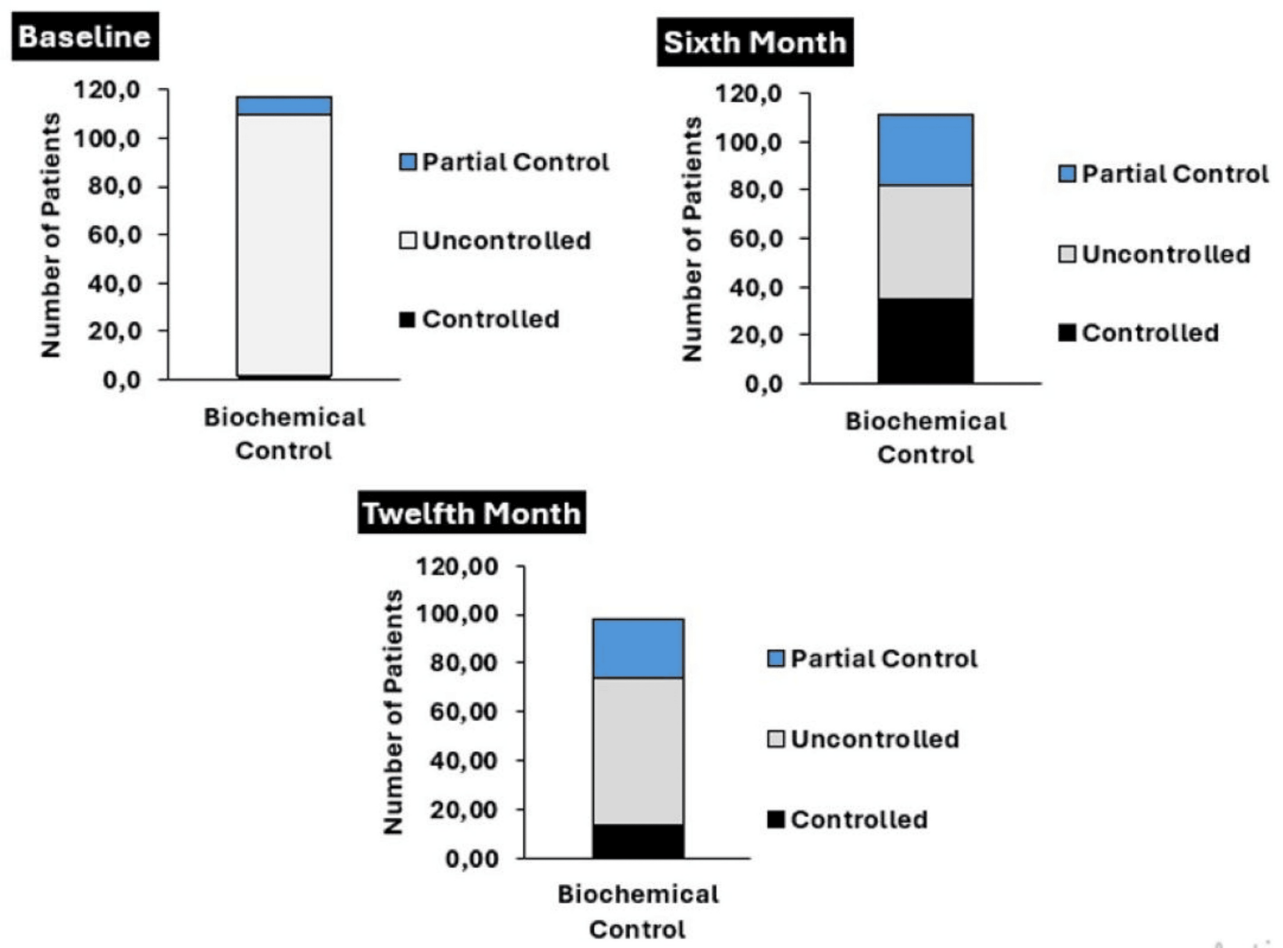
Global Biochemical Control Over Time

Biochemical control and tumor size were monitored at the time of entry into the registry, at six months and 12 months of follow-up, according to the active and cumulative interventions received in each period (Figure 2). At the beginning of the observation, n=108 (92.3%) of the patients had no biochemical control of the disease and n=7 (5.9%) had partial control. At that time, n=49 (41.9%) were managed only with surgical treatment, n=27 (23.1%) received drugs, and n=24 (20.8%) managed surgery and medications. A total of 17 (14.5%) subjects without any specific treatment at admission were documented. Of these groups, those managed with surgery were the largest subjects with biochemical control n/N=2/49 (4.4%) and partial disease control n/N=3/49 (6.1%), but without statistically significant differences in the first month of observation. Fifteen (12.8%) of the patients also underwent radiotherapy. 

**Figure 3:**
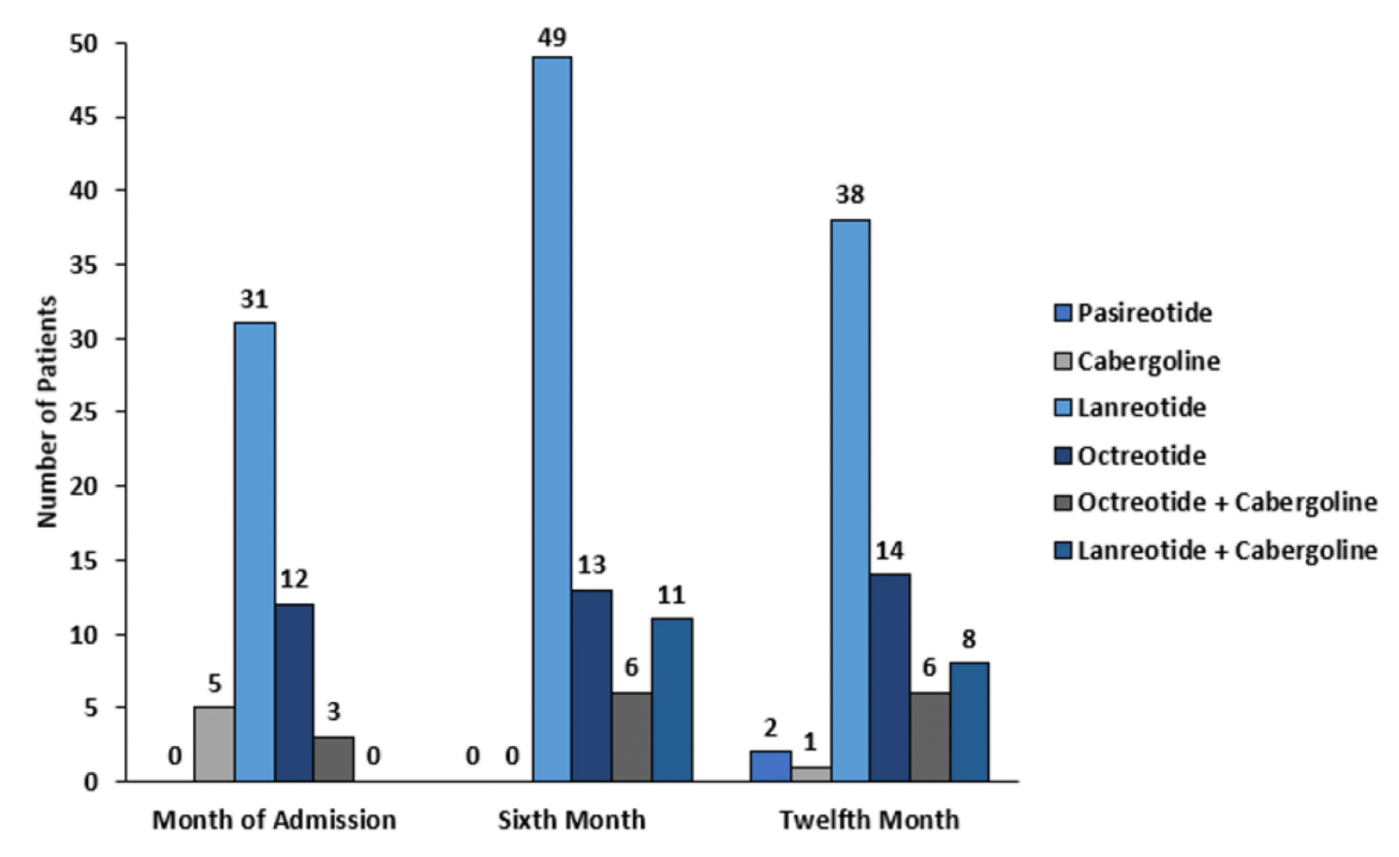
Description of the medications used in each month of observation

At month six, n=40 (34.1%) of the subjects achieved biochemical control, based on active intervention this control was observed mainly in the group managed only with medications n/N=24/61 (39.4%), followed by patients treated with surgery and medications n/N=5/18 (27.8%). The latter group also contributed the largest number of patients with partial biochemical control of the disease n/N=6/18 (33.3%). The proportion of uncontrolled subjects was significantly lower in the group of patients with only drugs, compared to the group with surgery alone (p=0.001). The control based on the interventions accumulated for this period, no statistically significant difference was observed between any of the groups. In this time period, the most commonly used drug was lanreotide, followed by octeotride and the combination of each with cabergoline (Figure 3). There were six patients without active intervention, five with biochemical control and one with partial control. Thirteen (11.1%) patients received radiation therapy during this time of observation.

By month 12, only n=25 (21.2%) of the total subjects had complete biochemical control, when evaluating the active interventions, all were located in the drug group n/N=14/66 (21.21%); in the drugs and surgery group, partial control was achieved by n/N=2/3(66.7%) of the patients, although this proportion was not statistically higher compared to that of the surgery-only group (p=0.067). Regarding control based on cumulative interventions, no statistically significant difference was found between the different groups evaluated. During this period, no patients received radiotherapy.

**Figure 4:**
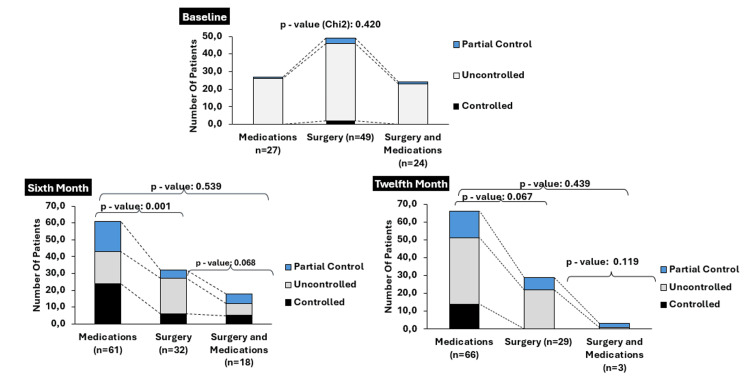
Description of the markers of partial biochemical control in each month of observation

In the three observation periods, partial control was always led by the IGF-1 biomarker (Figure 4). When the biochemical control categories were regrouped into controlled and non-controlled, it was found that the proportion of controlled was significantly higher at month six and month 12 when compared to the month of entry into the registry (p<0.001).

In total, 158 surgical procedures were performed on 103 patients. Thirty-nine (37.9%) of those operated on had to be reoperated once, while n=8 (7.8%) were reoperated on two occasions. There were no statistically significant differences between the number of surgical interventions and the biochemical control achieved (p=0.775).

Reduction in tumor size

**Figure 5:**
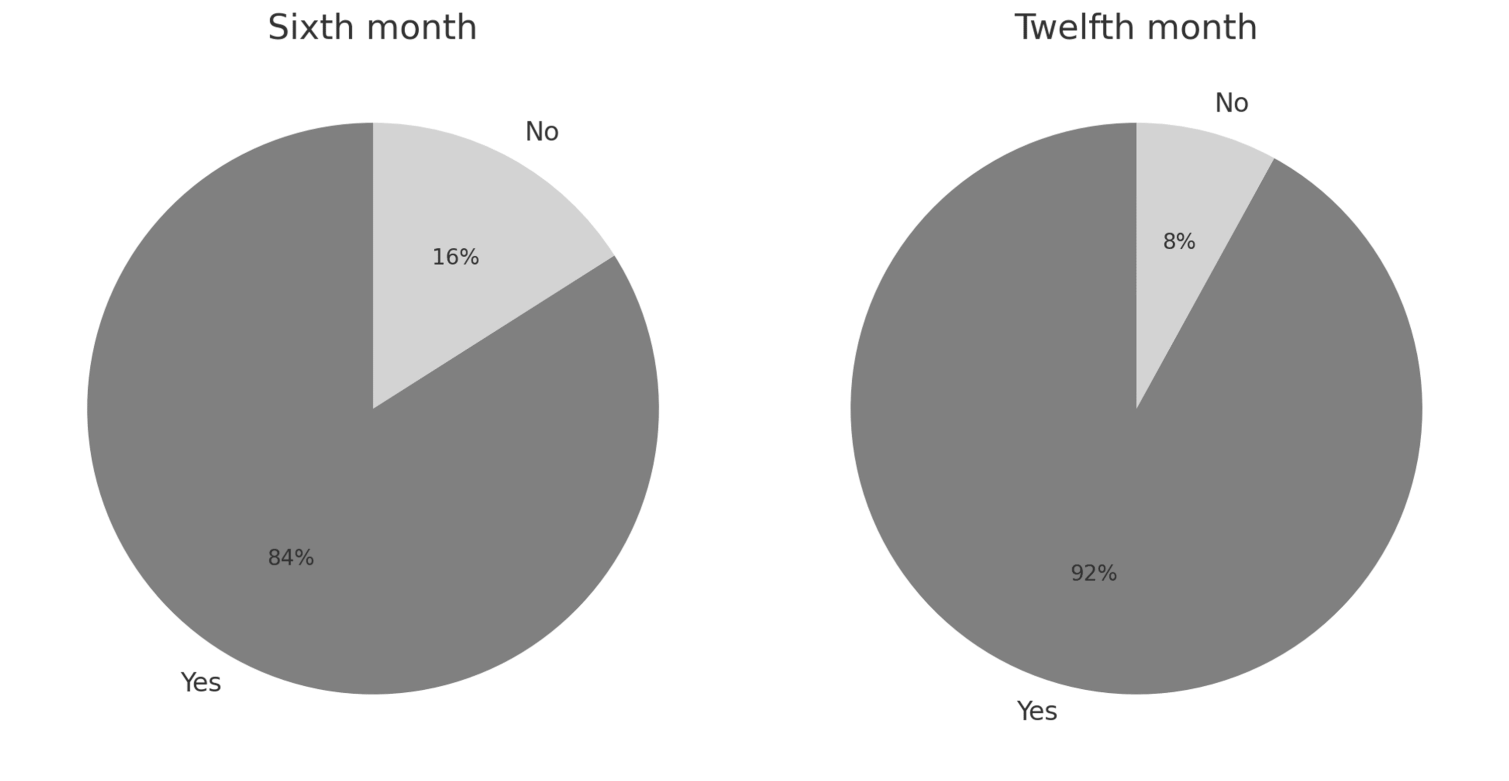
Tumor Size reduction > 20%

The median tumor size by MRI at the month of admission was 16 mm (IQR, 12 to 19). During the imaging follow-up, tumor size showed significant changes, with a greater than 20% reduction in the initial size in n/N=98/117 (84%) of patients at month six and n/N=108/117 (92%) at month 12. The percentage of tumor size reduction between the month of admission and month six was 50%, between the month of admission and month 12 it was 62.5%, and between months six and 12 it was 25% (p<0.001) (Figure 5).

## Discussion

The present study evaluated the biochemical control, types of treatment received, clinical characteristics, and histopathologies of pituitary adenomas in a Colombian registry of patients with acromegaly during a 12-month follow-up. At the beginning of follow-up, 99 (85%) of patients had undergone some type of intervention, the most common being surgery, which is considered the treatment of choice in acromegaly [6,11-12]. This intervention has been reported to achieve remission rates of up to 67% compared to pharmacological therapy (45%) [13]. The success of this depends on whether it is carried out in centers of experience in the management of pituitary pathology in charge of skull base neurosurgeons [14,15].

At the beginning of the observation, only 2.0 (1.7%) of the patients met the definition of biochemical control, even though most had already received some intervention, which is well below the control levels, regardless of the type of treatment [16]. If only the surgical intervention is evaluated at the beginning of the registry, 73 (62%) of the patients underwent previous surgery, and of these, only three (4.1%) had biochemical control, a situation that is lower than that reported in the literature, where biochemical control especially of IGF-1 is observed in 54.8% of the patients [17].

In the period evaluated, up to three out of four patients underwent surgery. At six months, almost half of the surgeries corresponded to first interventions, while at 12 months, approximately a quarter were. Biochemical control, after the first surgery, was reported in 12 (77%) of patients with microadenomas and 50 (57%) of patients with macroadenomas [17]. In those patients who undergo a new surgical intervention, biochemical control is expected in 67 (57%) of cases [18]. However, in this cohort, surgical patients did not achieve this control, regardless of the number of interventions received. 

Preoperative medical management is not usual in acromegaly, but at the six- and 12-month follow-up of the registry, patients with greater biochemical control based on active intervention received pharmacological management alone. However, when evaluating based on cumulative intervention, no statistically significant difference was observed in biochemical control. The meta-analysis by Nunes et al. (2015) collected data from four randomized studies and documented that preoperative use of SSA was associated with greater control of IGF-1 levels [19]. Although pharmacological management achieved the greatest biochemical control in the study population, the magnitude was lower than what was reported in the literature. SSAs have been shown to control GH levels by 56% and IGF-1 levels by 55% [19,20]. One possibility to impact and obtain better biochemical control with this type of medication is to increase the dose and/or frequency with which they are administered, as observed in the study by Giustina et al. (2017) [21]. 

Combination therapy was also not frequently used in the registry, being an alternative with evidence for the biochemical control of acromegaly [22]. The addition of dopamine agonists has been described mainly in patients with resistance to SSA use [23,24], being a useful strategy for biochemical control especially in patients with expression of PRL markers [25]. The registry showed that the association of progression-free survival (PFS) with dopamine agonists was low, and there was only one change of increase in this combination between months zero and six. This phenomenon may be related to therapeutic inertia or risk of adverse effects.

Pegvisomant was not used for the treatment of patients in the registry. This drug in monotherapy has a high effectiveness in achieving biochemical control and is a pharmacological combination option in association with SSA, with good cost-effectiveness in patients who have not achieved biochemical control with first-line strategies such as surgery or the use of SSA in monotherapy [26,27]. In addition, the transition from SSA to pegvisomant has a good safety profile, demonstrating to maintain control of tumor size and reduce hyperglycemia in SSAs [28,29]. However, it is still considered a second-line drug, the use of which must be individualized [30]. It is a treatment option to have in populations with poor control of the disease such as the one in the registry.

In the record, greater control of IGF-1 levels concerning GH was observed throughout the follow-up, especially at month six, which was the month with the highest global biochemical control. In general, GH levels are more of a tumor marker and IGF-1 levels are a marker of disease activity [31]. Although the follow-up aims to normalize both variables, a discrepancy between GH and IGF-1 control is observed in 13.7% to 35.4% of cases [32]; this is explained by the fact that IGF-1 levels reflect GH secretion over 24 hours, whereas an isolated GH measurement does not correlate with GH production over 24 hours [33]. Falch et al. (2023) in a registry of 178 patients with acromegaly followed for 12 months between 2005-2020 observed that IGF-1 normalization was a predictor of biochemical control in short-term surgical patients, they also found greater biochemical control in patients treated with drugs and surgery (82%) vs. surgery alone (53%) [34].

Regarding the impact of biochemical control based on tumor size reduction, the literature speaks of this particularly in the scenario of recurrence, with the most frequent being the elevation of IGF-1 or GH, but not both [35]. Tumor size reduction is still an important goal that has an impact on disease control and associated comorbidities [36]. This decrease has been established as significant when it is greater than 20% [11,12]. This reduction in the registry was observed in 95 (92%) of patients at 12 months, but this value of significant reduction is not standardized, and it is recommended to evaluate the reduction of the maximum diameter rather than the overall tumor volume [37]. In the registry, there was a significant decrease in the median diameter of tumor size with respect to baseline size at both six and 12 months. The impact of this on biochemical and comorbidity control is unclear [38]. In the population evaluated, despite a high rate of tumor size reduction, biochemical control was low, similar to data in the literature where it has been observed that patients with biochemical relapse, especially IGF-1 levels, had normal sella turcica MRI without tumor imaging relapse [39]. This indicates that the normalization of IGF-1 is more important, and suggests that, if biochemical control had already been achieved, follow-up should be only laboratory and imaging should be reserved for the IGF-1 relapser.

In acromegaly, macroadenoma is the most common tumor type, found in up to 75% of global population registries [40], like the 80% rate of macroadenomas observed in the analyzed registry. Histopathologically, tumors are GH producers [31]. The registry found that 60 (58.2%) of the patients had single GH expression. Expression of other markers from different cell lineages has also been reported, the most common being the association with PRL, and to a lesser extent other markers [41], similar to the findings observed in the registry. If we evaluate the size in diameter of the tumor, a median of 16 mm was obtained at the beginning of the record, these values are below the ranges in which there is commonly tumor invasion of the cavernous sinuses, a condition that is related to a lower probability of biochemical control [42]. There are also other markers of poor control of the disease, both molecular and histopathological, of which available in our setting we have the levels of Ki-67 [43], which has an impact on the possibility of recurrence, being higher when it is >3%, as well as being related to the risk of malignancy if it is >10% [44], in the registry we found a median of 2% in its value, with no significant difference between patients with microadenomas or macroadenomas, suggesting that it was not a factor associated with the low biochemical control of the registry.

In the literature, the mean age for the diagnosis of acromegaly, reported by Maione et al. (2019) in an analysis of 19 records worldwide was 45.2 years; in the population evaluated in the registry, we observed a higher median age, 52 years [40] with wide IQRs (40 to 60); contrasting with the Danish registry of Dal et al. (2016) in which the age at diagnosis was lower and with narrow intervals (48.7 years; 95% CI, 47.2 to 50.1) [45], reflecting lower recognition of the disease and greater limitations in access to health services in Colombia. The time of diagnosis of the disease is also important; periods of up to 10 years after the onset of symptoms have been described [46], with the mean observed in the Swedish registry of Esposito et al. (2020) being 5.5 years, although in up to a quarter of patients the delay can be greater than 10 years, which was related to higher morbidity and mortality [47]. In Colombia, Tovar et al. (2019) described an average evolution of symptoms to diagnosis of 8.3 years [48]. The registry did not have clear data on the time from the onset of symptoms to diagnosis, but a median time of disease evolution of six years was observed at the time of entry into the registry. 

In patients with acromegaly, assessment of comorbidities is as important as treatment that generates biochemical normalization [5,11,12]. There are multiple conditions associated with the disease, such as cardiovascular complications, diabetes or obstructive sleep apnea hypopnea syndrome (OSAHS), among others [49]. Arterial hypertension (HTN) is the comorbidity most associated with acromegaly [50] and was the most frequent in the population evaluated, a finding related to increased vascular resistance and expansion of vascular volume due to increased IGF-1 [51]. This comorbidity has been associated with a significant increase in mortality, especially if there are other cardiovascular diseases. The article evidenced that hypertension is associated with a 3.3-fold increase in mortality rate [52]. OSAS was the second most frequent comorbidity observed, being a condition with high prevalence in patients with acromegaly, related to increased mortality [53]. Its severity is positively influenced by treatment [54]. Heart disease secondary to the effect of IGF-1 on myocardial tissue generates multiple conditions such as left ventricular hypertrophy (LVH) and the development of arrhythmias, among others [55]. The prevalence of this entity is not clear, there are some records in which it is placed in up to 57% of patients, manifested mainly as LVH [56]. Arrhythmias associated especially with structural changes due to increased myocardial volume have been reported between 12 and 48% of acromegalic patients [57]. Both cardiomyopathy and arrhythmias were evidenced in a lesser proportion to global data, however, they are conditions that can increase morbidity and mortality [49]. Biochemical normalization, whether through surgical or pharmacological intervention, is related to improvement and even normalization of cardiac dysfunction, and impact on the patient's cardiovascular risk [58].

Acral growth is the most common manifestation for which acromegalic patients consult [59]. The prevalence of acral growth in men and women was the same without a statistically significant difference in the population analyzed. In addition, the registry observed that the median registry was in the range of overweight with higher IQR crossing the obesity range, which is associated with cardiovascular disease and complications [60].

With the publication of the most recent consensus on diagnostic criteria and remission of acromegaly, emphasis is placed on the use of standardized, endorsed, and calibrated tests; as well as treatment given by expert groups on the disease, to the greater relevance to biochemical control by IGF-1 levels, and the evaluation of the clinical activity of the disease [61]. In the population, there were no specific data on the calibration of the laboratory tests used, however, there was an adjustment of IGF-1 by age, and although the median BMI in the overweight range was observed in the population, which can generate lower IGF-1 values [62], its adjustment for this variable remains only optional. individualizing the patient [61]. GH measurement was mainly performed by ultrasensitive random test and the cut-off point of the most recent consensus <1 ng/ml) was used, but other strategies such as the oral glucose load tolerance test, which is also optional for follow-up, were not used [61]. The criteria for disease control continue to undergo changes and adjustments, giving greater importance to IGF-1 levels, which was observed in our population and was the most achieved biochemical parameter in the follow-up. However, as mentioned, the levels of control continued to be lower than described in the literature.

The main limitation of the study is its retrospective design due to the risk of information bias, since the data were obtained from patient registries, there were also no data in the recording of clinical variables after the month of admission, which limited the analysis of the effect of biochemical control on them. Among the strengths are the analysis of a large cohort of patients with acromegaly for 12 months, evaluating the biochemical control based on the therapeutic interventions received; the immunohistochemical characteristics of all tumors resected during follow-up are also described.

## Conclusions

In Colombian patients with acromegaly, biochemical control at 12 months is lower than that reported in the literature. Pharmacological management as an active intervention could achieve higher control rates. The reduction in tumor size evaluated with current standards is achieved in a large proportion of patients, however, it does not impact biochemical control. The results of this study suggest that in our setting, where access to centers of excellence in pituitary surgery is unknown, the use of medications may have greater relevance in achieving biochemical control.
